# Cloning and overexpression of *PeWRKY31* from *Populus* × *euramericana* enhances salt and biological tolerance in transgenic *Nicotiana*

**DOI:** 10.1186/s12870-021-02856-3

**Published:** 2021-02-06

**Authors:** Xiaoyue Yu, Yu Pan, Yan Dong, Bin Lu, Chao Zhang, Minsheng Yang, Lihui Zuo

**Affiliations:** 1 Forest Department, Forestry College, Hebei Agricultural University, Baoding, China; 2 Hebei Key Laboratory for Tree Genetic Resources and Forest Protection, 071000 Baoding, P. R. China; 3 Tianjin nuohe medical laboratory co. LTD, Tianjin, China; 4 College of Landscape Architecture and Tourism, Hebei Agricultural University, Baoding, China; 5 College of Landscape and Ecological Engineering, Hebei University of Engineering, 056000 Handan, P. R. China

**Keywords:** WRKY, Genetic transformation, Salt tolerance, Insect resistance, Transcriptome

## Abstract

**Background:**

As important forest tree species, biological stress and soil salinization are important factors that restrict the growth of *Populus × euramericana*. WRKYs are important transcription factors in plants that can regulate plant responses to biotic and abiotic stresses. In this study, *PeWRKY31* was isolated from *Populus × euramericana*, and its bioinformation, salt resistance and insect resistance were analyzed. This study aims to provide guidance for producing salt-resistant and insect-resistant poplars.

**Results:**

*PeWRKY31* has a predicted open reading frame (ORF) of 1842 bp that encodes 613 amino acids. The predicted protein is the unstable, acidic, and hydrophilic protein with a molecular weight of 66.34 kDa, and it has numerous potential phosphorylation sites, chiefly on serines and threonines. PeWRKY31 is a zinc-finger C_2_H_2_ type-II WRKY TF that is closely related to WRKY TFs of *Populus tomentosa*, and localizes to the nucleus. A *PeWRKY3*1 overexpression vector was constructed and transformed into *Nicotiana tabacum* L. Overexpression of *PeWRKY31* improved the salt tolerance and insect resistance of the transgenic tobacco. Transcriptome sequencing and KEGG enrichment analysis showed the elevated expression of genes related to glutathione metabolism, plant hormone signal transduction, and MAPK signaling pathways, the functions of which were important in plant salt tolerance and insect resistance in the overexpressing tobacco line.

**Conclusions:**

*PeWRKY31* was isolated from *Populus × euramericana*. Overexpression of *PeWRKY31* improved the resistance of transgenic plant to salt stress and pest stress. The study provides references for the generation of stress-resistant lines with potentially great economic benefit.

**Supplementary Information:**

The online version contains supplementary material available at 10.1186/s12870-021-02856-3.

## Background

A wide variety of plant transcription factors interact with defense-related gene promoters, ultimately enhancing plant resistance to stress [1]. The transcriptional regulation of stress response genes not only helps plants respond to various stressors [2], but is also important for plant growth, development, and metabolic pathways [3]. Transcription factors, present in various families with complex functions, play an important role in plant development, metabolism, and responses to biotic and abiotic stresses. Transcription factors such as NAC, AP2/ERF, and WRKY play important roles in the regulation of plant growth under stress [4].

WRKYs form one of the largest transcription factor families, with the seventh-highest numbers among the higher plant transcription factor families, behind bHLH, MYB, ERF, NAC, C_2_H_2_, and bZIP [5]. WRKYs have been identified in 165 plants, a total of 14,549 of which are predicted to be involved in responses to biological and abiotic stresses, the transmission of hormone signals, and the regulation of growth and development [6, 7]. In abiotic stress, WRKYs mainly function in stresses caused by cold and heat [8], drought and flood [9–11], high salt [12–16], and excess radiation [17]. WRKYs also play crucial roles in plant immune responses to biological stress, defense responses to pathogens and bacteria [18–20], and in establishing relevant signal transduction pathways [21, 22]. The WRKYs of *Arabidopsis* and rice have been shown to play a role in insect resistance [23, 24], but few studies have investigated poplar WRKYs in the context of insect resistance.

Poplar species are commonly used as model woody plants due to their rapid growth and reproduction, wide distribution, and high adaptability. Additionally, poplar species are commercially important in China. Among them, *Populus × euramericana* breeds easily, grows fast and has strong adaptability. In recent years, with the completion of the sequencing of the *Populus trichocarpa* genome and the continuous development of biotechnology tools, poplar research has been more focused on the molecular level. The latest release of the PlantTFDB (http://planttfdb.cbi.pku.edu.cn/)transcription factor database contains a total of 185 *Populus tomentosa* WRKYs. Over a dozen related transcription factors have been isolated, their functions have been verified [25], and the WRKY family genes in many poplar trees have been shown to respond to abiotic stress and play important roles in salt resistance [26–28]. Preliminary laboratory research revealed that *PeWRKY3*1 of *Populus × euramericana* responded to biological stress, but the underlying mechanism was not clear. In order to clarify the function, *PeWRKY31* was isolated from *Populus × euramericana* and its sequence was analyzed using bioinformatics in this study. We constructed a vector for subcellular localization analysis and a vector for tobacco overexpression. Then, we identified the role of PeWRKY31 in signal transduction pathways responding to salt and pest stress. The research lays a foundation for the generation of stress-resistant *Populus × euramericana* lines.

## Results

### Screening and cloning of *PeWRKY31*

Total RNA was extracted from leaves of *Populus × euramericana* clones and lines that had been treated with 4% salt or chewed by *Clostera anachoreta*. The OD_260_/OD_280_ was between 1.8 and 2.0, and the OD_260_/OD_230_ was greater than 2.0, indicating that the total RNA was of sufficient purity. cDNA was used as a template for determination of *PeWRKY31* expression and PCR with the primer set 31#all F/R (Table S1). The target gene *PeWRKY31* was amplified by PCR using 31# All F/R as primer (Table S1). The resulting PCR product was about 1800 bp, consistent with the expected size, and was used for carrier construction.

**Fig. 1:**
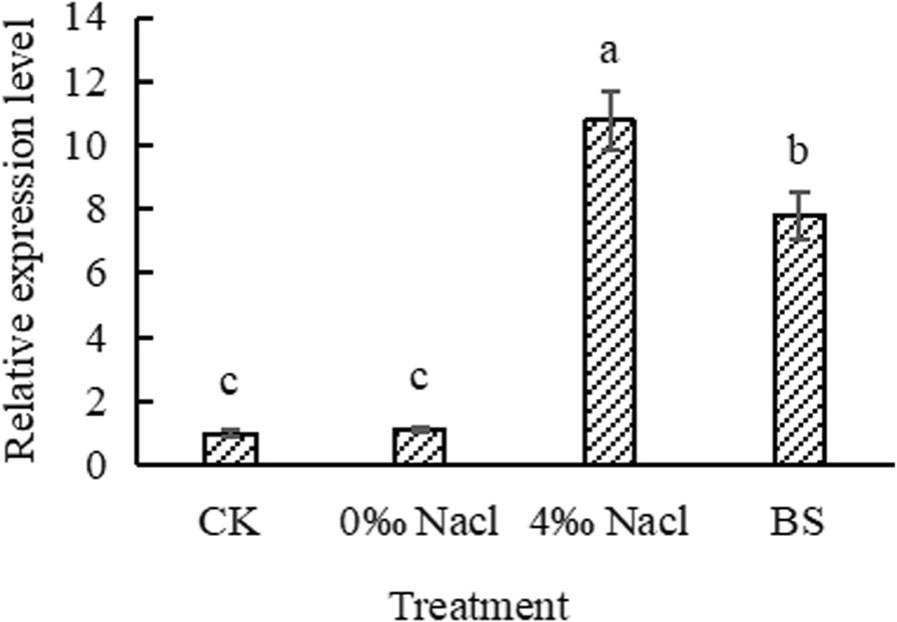
Relative expression of *PeWRKY31* under different treatments. BS: Insects stress. The means ± SD of three biological replicates are presented. Error bars represent the standard deviation of the mean. Different letters above the bars indicate a statistically significant at the *P* < 0.05 level according to LSD multiple range test

To analyze the expression of *PeWRKY31* in *Populus × euramericana* under different stress, *Populus × euramericana* was treated with 4‰ salt and larval feeding. The RT-PCR results showed that the expression of *PeWRKY31* changed after treatment with 4‰ NaCl for 7 days and larval feeding for 1 h. The relative expression of *PeWRKY31* increased by about 10 times and 8 times, respectively, after 4‰ NaCl treatment and biological stress (Fig. 1), indicating that biological and abiotic stress regulated *PeWRKY31* expression.

### Bioinformatics analysis of *PeWRKY31*

The full-length predicted ORF of *PeWRKY31* comprises 1842 bp that encodes 613 amino acids. The predicted protein has a molecular mass of 66.34 kDa, an isoelectric point of 6.12 (acidic), an instability coefficient of 48.59, and a hydrophobicity of − 0.712. These findings indicate that PeWRKY31 is an acidic and unstable hydrophilic protein. In the secondary structure of PeWRKY31, α-helix accounted for 24.96%, β-rotation for 2.94%, extension chain for 12.56%, and random structures for 59.54% (Fig. S1-A). The tertiary structure model was mainly composed of five β-fold structures and random structures (Fig. S1-B). cNLS analysis showed that PeWRKY31 contained the nuclear localization signal sequence PSDNRRR, suggesting that the protein was located on or in the nucleus. Since no predicted transmembrane region was found and there was no predicted signal peptid, the protein is likely intracellular (Fig. S2-A). The protein had a total of 75 potential phosphorylation sites, dominated by serine and followed by threonine.

**Fig. 2:**
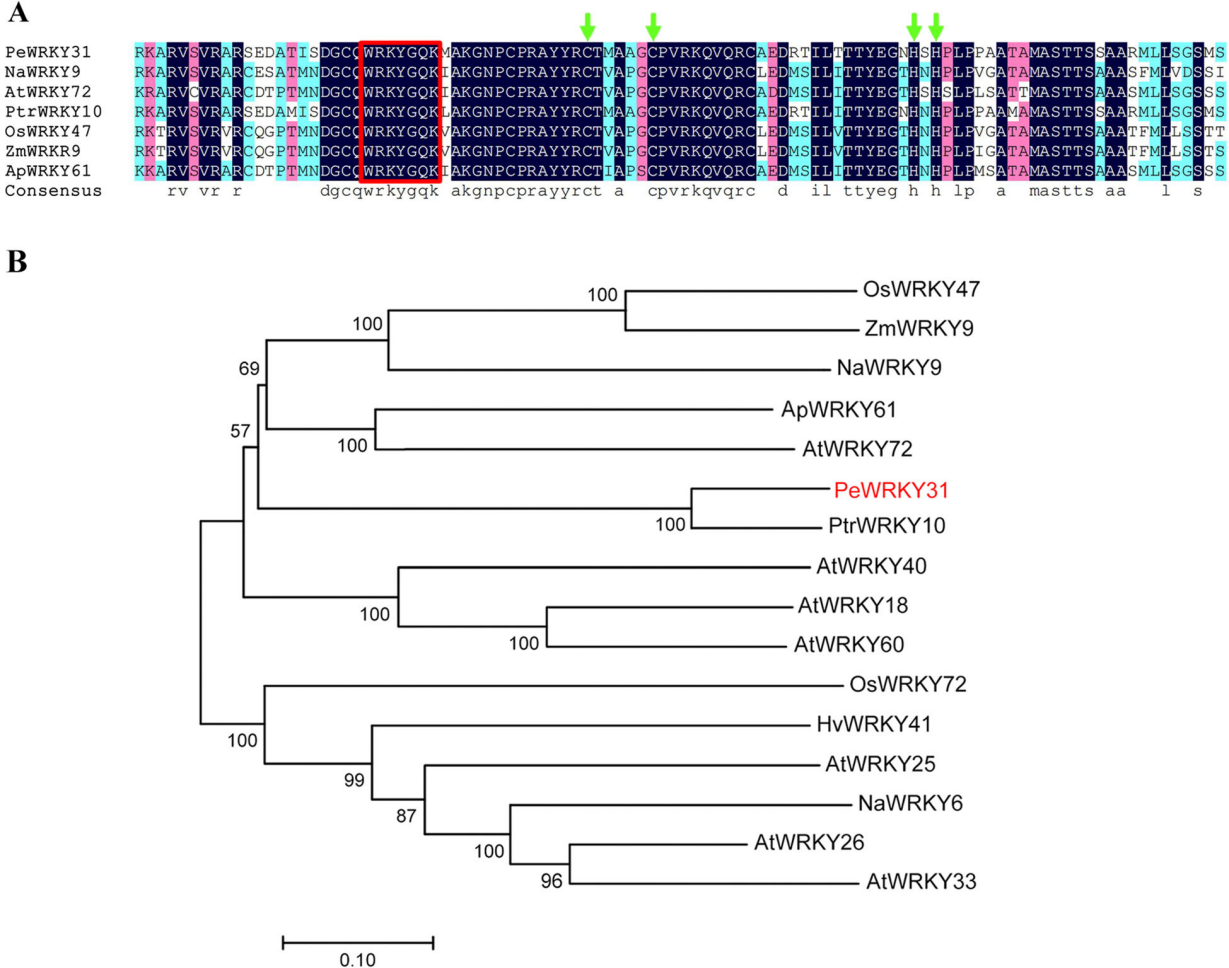
Alignment of sequences and phylogenetic tree of the WRKY proteins. **a:** Alignment of sequences of the WRKY proteins PeWRKY31 and homologs from other species. Red box indicates the WRKYGQK conserved sequence, and green arrow indicates the C_2_H_2_ zinc-finger structural motif. **b:** Phylogenetic tree of WRKY proteins from *Populus × euramericana* and other species. Na, *Nicotiana attenuate*; At, *Arabidopsis thaliana*; Ptr, *Populus trichocarpa*; Os, *Oryza sativa*; Zm, *Zea mays*; Ap, *Amygdalus persica*; Hv, *Hordeum vulgare*

The amino acid sequences were most closely related to *PeWRKY31* in *Nicotiana attenuata*, *Arabidopsis thaliana*, *Populus trichocarpa*, *Oryza sativa, Zea mays*, and *Amygdalus persica* were compared using BlastX (https://blast.ncbi.nlm.nih.gov/Blast.cgi), Phytozome v12.1 (https://phytozome.jgi.doe.gov/pz/portal.html), and DNAMAN (https://www.lynnon.com/dnaman.html) software (Fig. 2a). All seven proteins contained a WRKYGQK sequence and a C_2_H_2_ zinc-finger structure, which were the typical characteristics of Group II WRKY proteins, indicating that they belonged to the class-II WRKY subfamily. *PeWRKY31* showed high similarity in WRKY domain compared with WRKY proteins of other species.

Searches were conducted in the NCBI and Phytozome databases (v12.1) using sequences of the *PeWRKY31* gene and 15 homologs from seven species (*Oryza sativa*, *Zea mays*, *Nicotiana attenuata*, *Amygdalus persica*, *Arabidopsis thaliana*, *Populus trichocarpa*, and *Hordeum vulgare*). The evolution of genes from different species was analyzed using the neighbor-joining method in MEGA ver. 7.0 (https://www.megasoftware.net) [25], and a phylogenetic tree was constructed using the results (Fig. 2b). *PtrWRKY10* had the closest genetic relationship to *PeWRKY31*, followed by *AtWRKY72*. The function of *PtrWRKY10* was unkonwn, but its closely related *AtWRKY72* responded to abiotic stresses such as salt injury [29]. Thus, *PeWRKY31* may also respond to salt stress.

### Subcellular localization of *GFP-PeWRKY31* in transiently transformed tobacco

**Fig. 3:**
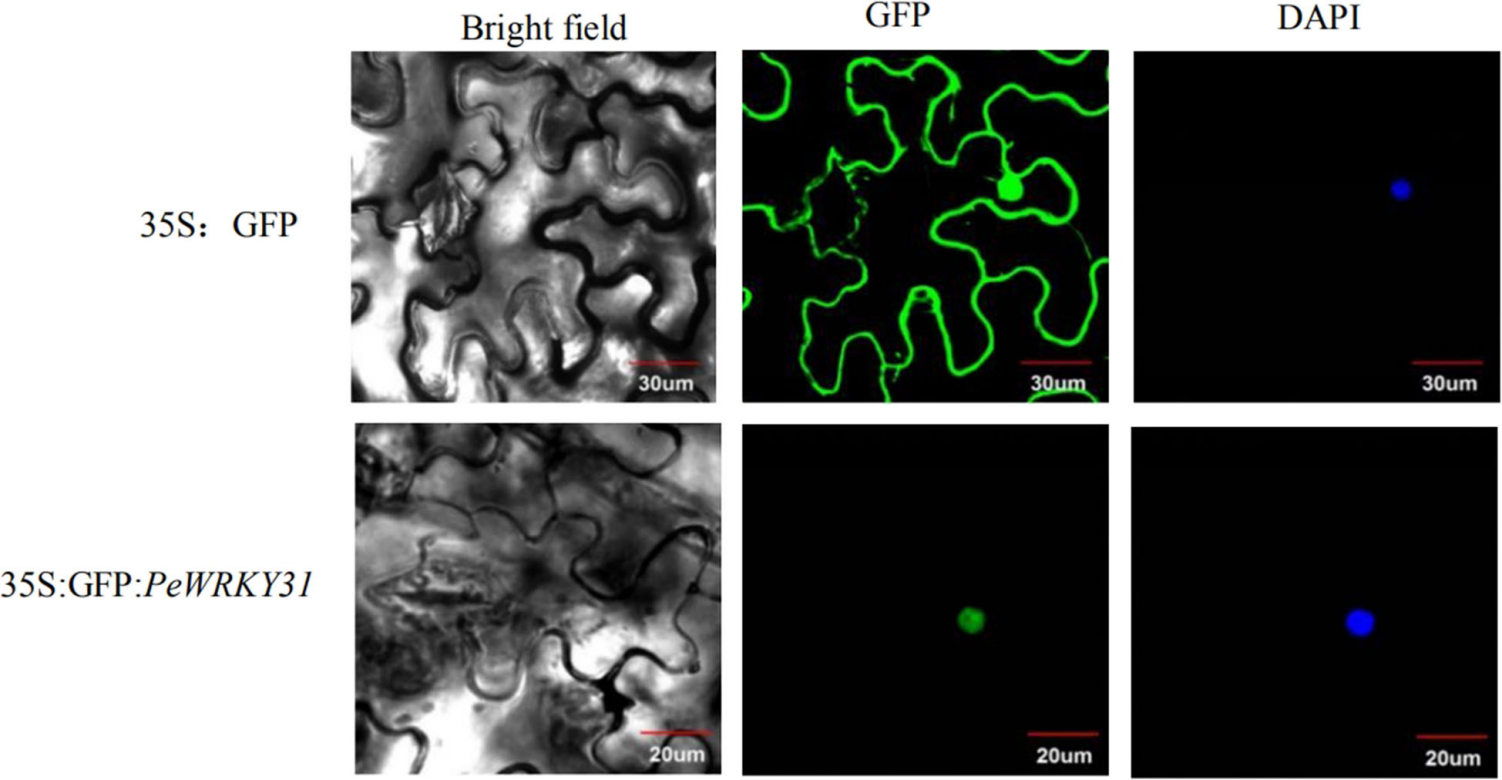
Subcellular localization of *PeWRKY31*

The fusion expression vector 35S:GFP-*PeWRKY31* (p31#subl+ 1302) and the control vector 35S:GFP were transiently transformed into tobacco leaves using the corresponding *Agrobacterium tumefaciens* strains by bacterial infection. The leaves were then observed using a fluorescence microscope (Fig. 3). DAPI fluorescent dye used as a control revealed the locations of nuclei. In leaves transformed with the 35S:GFP vector, the green fluorescent GFP signal was distributed in the nucleus and cytoplasm, indicating non-specific localization, as expected. In leaves transformed with 35S:GFP-*PeWRKY31*, the GFP fluorescence signal appeared inside cells. Only nuclei showed green fluorescence; no fluorescence signal was detected in the cytoplasm outside nuclei, indicating that GFP-PeWRKY31 localized specifically in the nuclei of tobacco leaves. These results show that PeWRKY31 is a nuclear-localized protein.

### Generation of transgenic tobacco lines

**Fig. 4:**
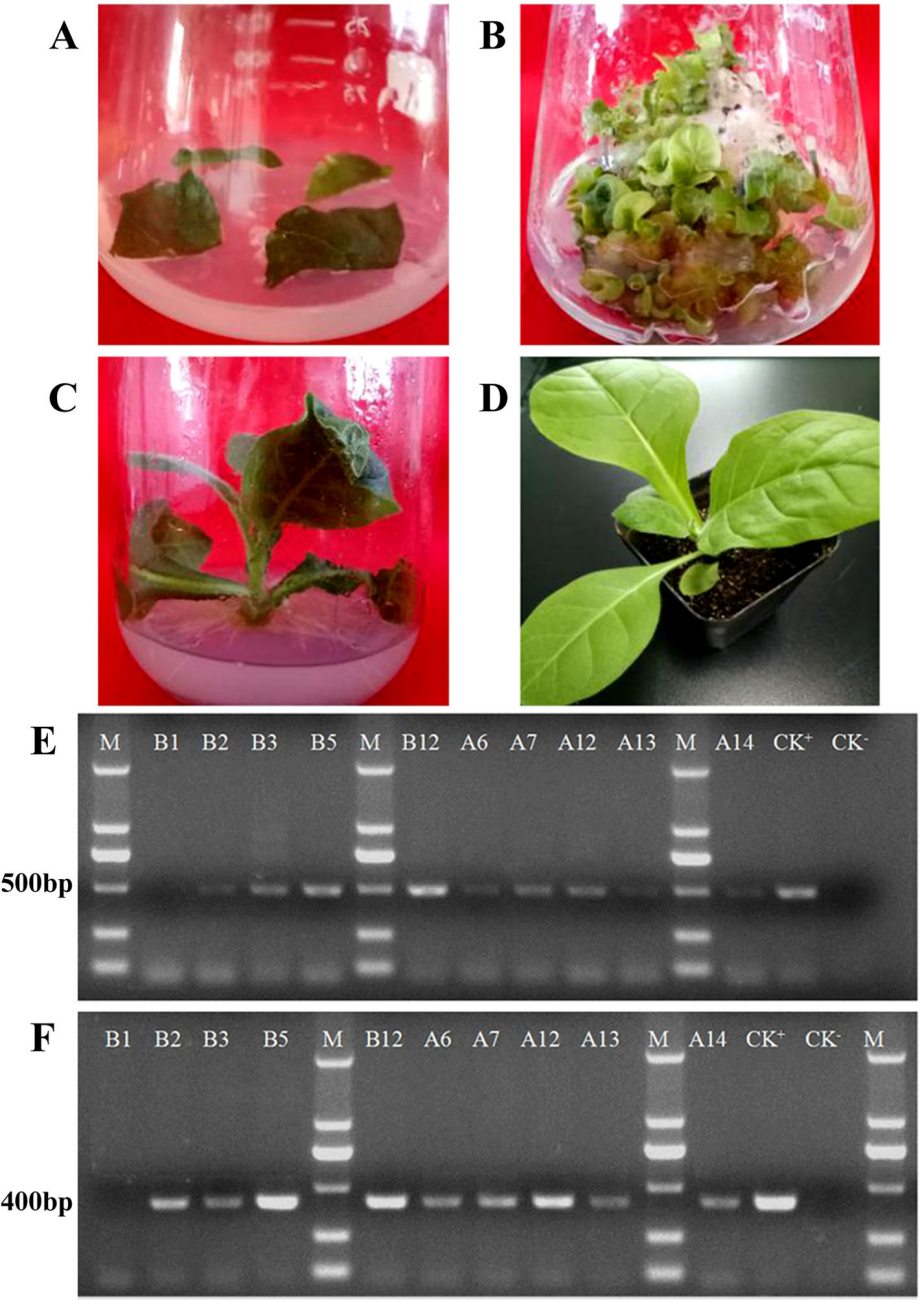
Generation of *PeWRKY31*-overexpressing transgenic tobacco and PCR analysis of the ten transformed tobacco lines. **a:** Leaves used for *Agrobacterium* infection. **b:** Screening for resistant vegetative propagules. **c:** Rooted plantlet. **d:** Transplanted plantlet. **e:** *nptII* gene fragment (~ 500 bp) amplified from transgenic tobacco. **f:** Portion of *PeWRKY31* gene (~ 400 bp) amplified from transgenic tobacco. Lane M, DL2000 DNA marker of US EVERBRIGHT; other lanes, test samples

The overexpression vector p31#op+ 121(Fig. S3B) was transformed into tobacco using the *Agrobacterium tumefaciens*-mediated leaf disk method. After 36 h of co-culture followed by screening for resistant vegetative propagules, green resistant vegetative propagules were transferred into rooting medium. Rooted tobacco plantlets were transferred into pots and allowed to grow under normal greenhouse conditions (Fig. 4a-d).

The DNA of ten tobacco lines was subjected to PCR using the specific primers 103#F/R, which amplified an *nptII* fragment of approximately 500 bp (Fig. 4e), and 35#send F and 31#-JC R, which amplified a ~ 400-bp portion of *PeWRKY31*(Fig. 4f) (Table S1 for all primer information). The plasmid containing the target gene was used as a positive control (CK+) and WT tobacco was used as a negative control (CK-). The target fragments were amplified from nine of the ten tobacco lines (except lane 1) and the positive control, but not the negative control. Thus, nine of the ten tested tobacco lines were confirmed to contain the target construct.

**Fig. 5:**
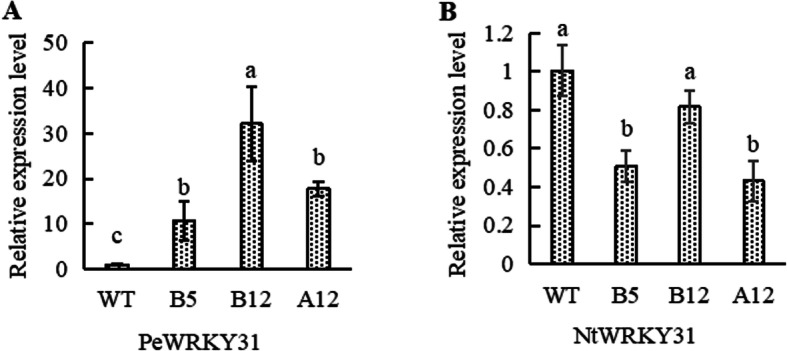
Relative expression level of qRT-PCR. **a:** Relative expression of *PeWRKY31* in different transgenic lines. **b:** Relative expression of *NtWRKY31* in different transgenic lines. The means ± SD of three biological replicates are presented. Error bars represent the standard deviation of the mean. Different letters above the bars indicate a statistically significant at the *P* < 0.05 level according to LSD multiple range test

The qRT-RCR results showed that the relative expression of *PeWRKY31* in transgenic lines B5, B12, and A12 was significantly higher than that in the WT, by 10, 31 and 17 times, respectively (Fig. 5a). The relative expression of the endogenous homologous gene *NtWRKY31* (LOC107770788) was significantly lower in transgenic lines B5 and A12 than in the WT (Fig. 5b).

### Salt tolerance analysis of transgenic tobacco overexpressing *PeWRKY31*

**Fig. 6:**
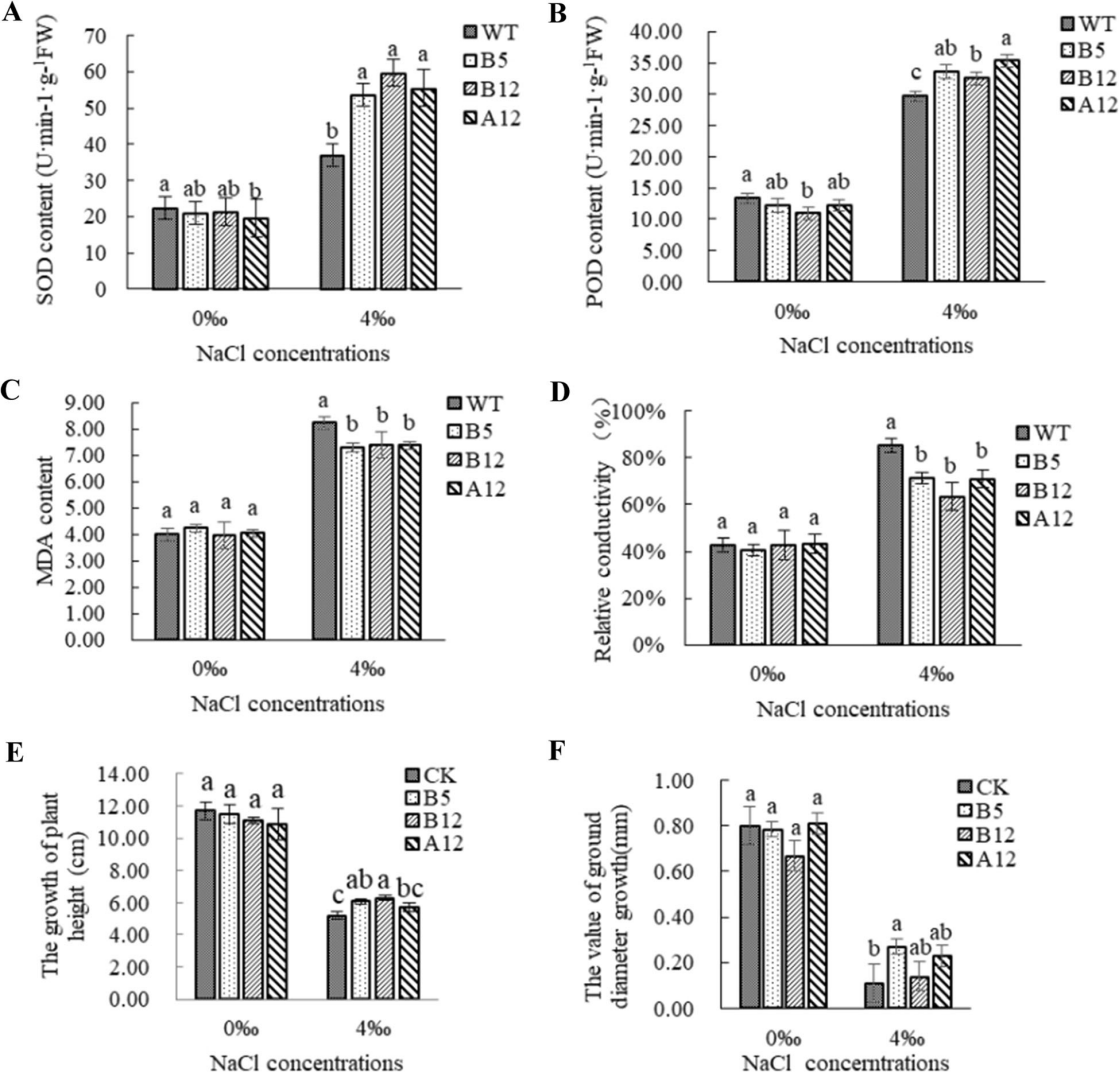
Effect of salt stress on *PeWRKY31*-overexpressed tobacco. **a:** SOD, **b:** POD, and **c:** MDA contents under NaCl stress. Variation in the **d:** relative conductivity, **e:** plant height, and **f:** ground diameter. The means ± SD of three biological replicates are presented. Error bars represent the standard deviation of the mean. Different letters above the bars indicate a statistically significant difference between the transgenic lines at the *P* < 0.05 level according to LSD multiple range test

The activities of the antioxidant enzymes SOD and POD in plants reflect the degree of stress damage to plants. The SOD and POD activities in the WT and transgenic tobacco lines were lower after 72 h of 0‰ salt stress (Fig. 6). In the presence of a 4‰ salt solution, the SOD and POD activities were increased in all lines wheras were significantly higher in the transgenic lines than in WT plants.

The MDA content and electrical conductivity reflect the integrity of the cell membrane. After 72 h of salt stress, there was no difference in the MDA content or the relative conductivity in WT tobacco and the transgenic lines under 0‰ salt concentration. In the presence of 4‰ salt, the MDA content of each line increased significantly, and the relative conductivity increased from 40% to 60–80% compared to the treatment without salt stress. The MDA content and relative conductivity of the transgenic lines were significantly lower than those of the WT, but there was no difference in the results among the transgenic lines. These results showed that salt stress caused oxidative damage to the cell membranes of all of the tobacco lines, and the cell membrane damage in the WT was more serious than in any of the transgenic strains. Therefore, the target gene had a protective effect on the cytoplasmic membranes and improved the salt tolerance of tobacco.

The plant height and ground diameter of WT tobacco and the transgenic lines were monitored throughout the 5-day period (Fig. 6). The plant height growth rates of all tobacco lines decreased significantly under salt stress; the growth in height and the ground diameter growth of the transgenic lines, especially lines B12, were significantly higher than that of the WT, indicating that the transgenic lines had more robust growth than the WT under salt stress. This indicated that the overexpression of *PeWRKY31* improved the salt tolerance of tobacco.

### Insect resistance analysis of *PeWRKY31*-overexpressing transgenic tobacco

**Fig. 7:**
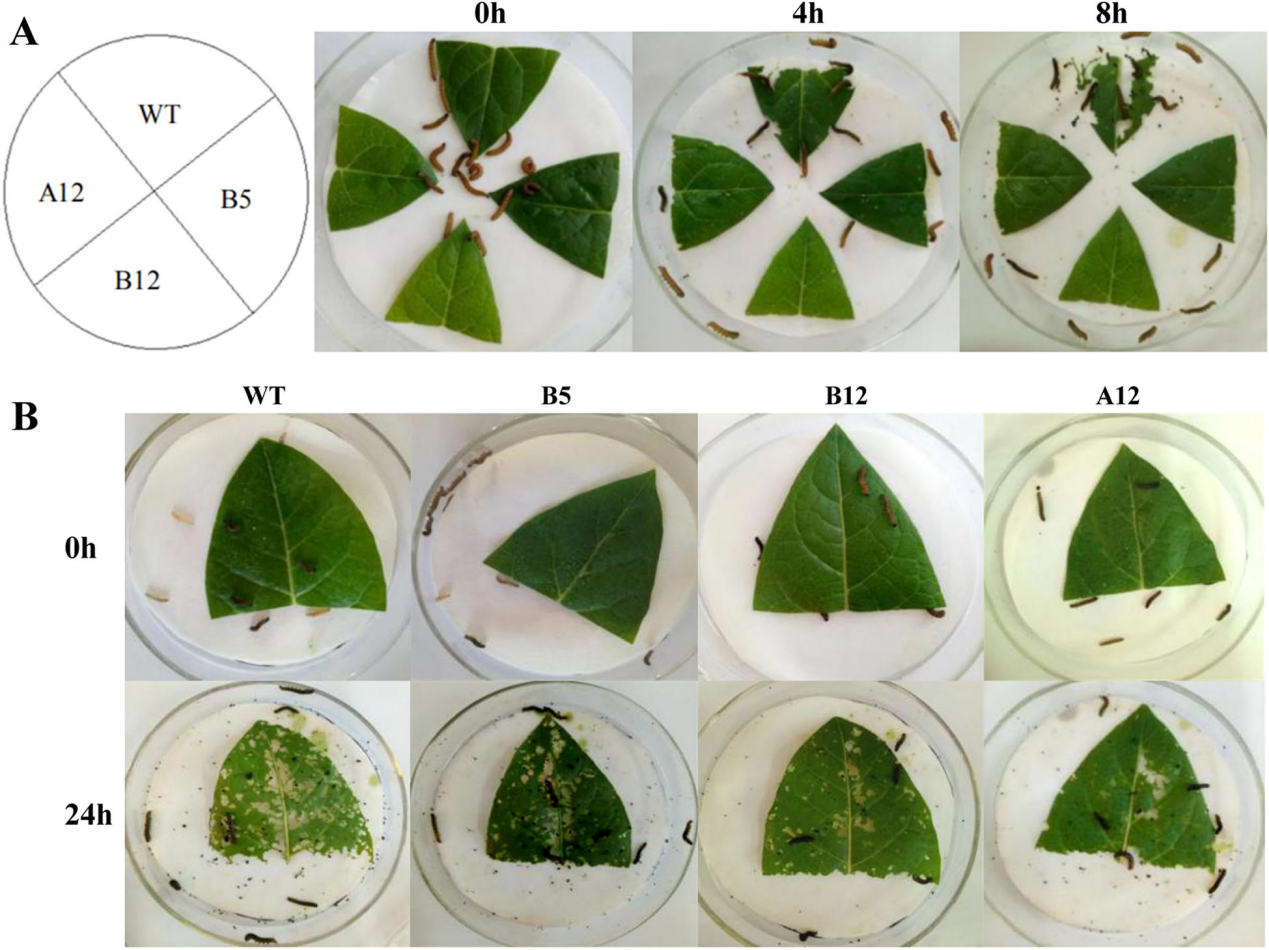
Feeding experiments of cotton bollworm on *PeWRKY31*-overexpressing transgenic tobacco. **a:** Feeding preference (choice or preference). **b:** Mandatory feeding (no choice)

Leaves of WT tobacco and the transgenic lines of similar area and equal quality were placed in the same petri dish to observe the feeding preference of cotton bollworms (Fig. 7a). After feeding for 4 h, it was evident that the insects had focused mainly on WT tobacco leaves, as higher levels of insect chewing damage were visible. After 8 h of feeding, the area of leaf loss of WT tobacco was nearly 50%, whereas that of all of the transgenic lines was below 10%. These results indicated that *PeWRKY31* improved the insect resistance of tobacco.

Leaves of WT tobacco and the transgenic lines of similar area and equal quality were placed in separate petri dishes with cotton bollworms, and the leaf loss was observed after 24 h (Fig. 7b). The leaf area loss of WT tobacco was greater than that of the transgenic lines, and the leaf losses of the B12 and A12 transgenic lines were less than that of the B5 line, indicating that the B12 and A12 lines had greater insect resistance.

### Transcriptome analysis of *PeWRKY31*-overexpressing transgenic tobacco

WT tobacco and the B12 transgenic tobacco line overexpressing *PeWRKY31* were subjected to transcriptome sequencing. 37,628 and 38,271 expressed genes were detected from the transgenic and WT tobacco lines, respectively, and 1696 unique genes were detected in transgenic tobacco, whereas 2339 unique genes were detected in WT tobacco. The number of differentially expressed genes in transgenic and WT tobacco was 14,892, with 6978 upregulated genes and 7914 downregulated genes in the transgenic line (Fig. S4-B).

Gene ontology functional classification of genes was used to examine the functional categories of the differentially expressed genes. Among all of the differentially expressed genes, 4753 were annotated to 677 biological processes, 1635 were annotated to 177 cellular components, and 6704 were annotated to 457 molecular functions, among which the differentially expressed genes enriched 44 biological processes, seven cellular components, and 27 molecular functions. The biological processes comprised mainly metabolic processes, the main cellular components were thylakoid and photosystem, and the main molecular functions were catalytic activity and binding (Fig. S4-C).

In addition, the KEGG database was used to analyze the enrichment of the differentially expressed genes in different pathways. 1233 differentially expressed genes of *PeWRKY31*-overexpressing transgenic tobacco were annotated to 113 specific metabolic pathways. The pathways include those involved in plant hormone signal transduction, plant-pathogen interactions, plant MAPK signaling pathways, carotenoid biosynthesis, terpenoid and ketone biosynthesis, ascorbate metabolism, glutathione metabolism, ubiquinone, and biosynthesis of other terpenoids and quinones. Phenylpropanoid biosynthesis and other pathways listed above are correlated with plant disease, insect resistance, and salt resistance.

In the *PeWRKY31*-overexpressing transgenic line, the expression of genes related to stress was significantly upregulated. In the glutathione metabolism pathway, the expression of *GSH-Px* was significantly upregulated. Ascorbate peroxidase (APX), whose gene was also upregulated, scavenges H_2_O_2_ in plants, especially in chloroplasts. In plant hormone signal transduction, the expression of *JAR1* was upregulated. The upregulated expression of *NPR1* and the binding of TGA transcription factors to the *NPR1* promoter can initiate broad-spectrum resistance in tobacco. Among the plant MAPK signaling pathways, there were two key pathways related to disease resistance, two related to wounding, and two related to cold resistance, drought resistance, and saline-alkali tolerance. In the wounding pathway, the expression of *RbohD* and *OXI1* were upregulated. In plant MAPK signaling pathway, MAPK5, MAPK8, MAPK1, MPK2 and MPK6, which were related to abiotic stress, were upregulated.

**Fig. 8:**
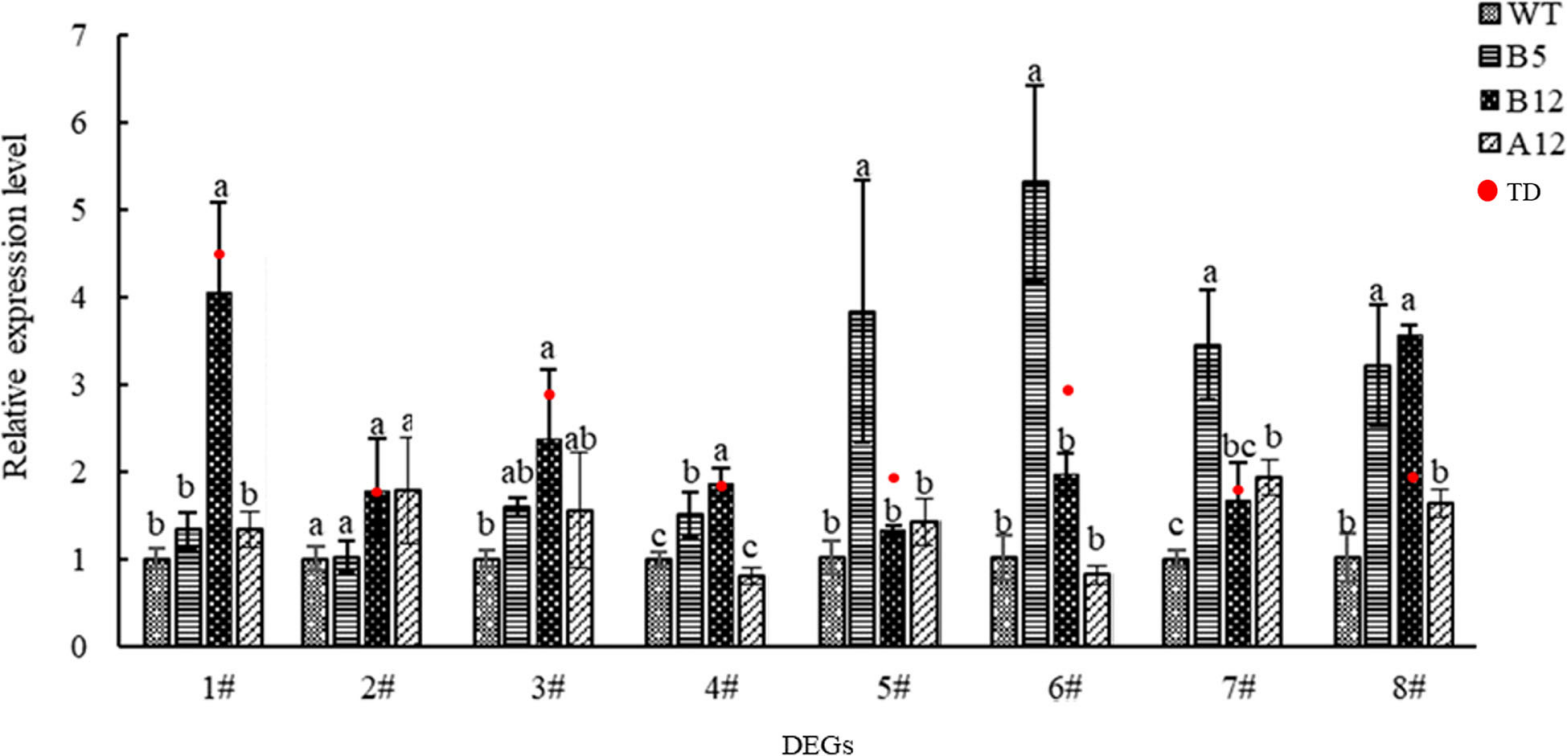
Relative expression of selected DEGs, compared using qRT-PCR and RNA-Seq. 1#: LOC107782952; 2#: LOC107798207; 3#: LOC107831218; 4#: LOC107768362; 5#: LOC107824233; 6#: LOC107832641; 7#: LOC107770091; 8#: LOC107816761; TD: Relative expression level of RNA-seq result. “TD” refers to the ratio of gene expression between the treatment and control groups. After treatmented with difference analysis software shrinkage model, the final logarithm was taken as the base of 2, namely log_2_(Fold Change). The means ± SD of three biological replicates are presented. Error bars represent the standard deviation of the mean. Different letters above the bars indicate a statistically significant at the *P* < 0.05 level according to LSD multiple range test

To verify the reliability of the differentially expressed genes (DEGs) results, eight upregulated DEGs related to biological and abiotic stress were selected for quantitative reverse transcriptase–polymerase chain reaction (qRT-PCR) verification, with *NtActin* used as the internal reference gene. Figure 8 shows the sequencing results of the eight genes and the qRT-PCR results. Although the qRT-PCR gene expression results for line B12 were not completely consistent with the sequencing results, the expression patterns of all eight genes used for verification were basically consistent with the DEGs, which showed the accuracy and credibility of the sequencing data. The differences in relative expression of DEGs among different transgenic lines might be caused by different insertion locations, and their expression patterns will also differ by insertion location (see Table S1 for the primer sequences).

## Discussion

### Subcellular localization analysis of *PeWRKY31*

Growing transcriptome databases resulting from continuously developing bioinformatics technologies and high-throughput sequencing projects are increasingly being used for gene mining and functional research [30], and PCR methods are used to obtain the target genes [31]. In this study, qRT-PCR showed that *PeWRKY3*1 responded to biotic and abiotic stresses.

Most activities of WRKY proteins take place in the nucleus. WRKY proteins of *Arabidopsis* [32] and rice [33] localizes to the nucleus, and fluorescent signals from fusion proteins of *PtrWRKY40* in *Populus trichocarpa* and *PtoWRKY60* in *Populus tomentosa* are observed exclusively in the nucleus [34]. In addition, the *OfWRKY3*,*CaWRKY6* and *CaWRKY30* proteins are also located in the nucleus [35]. Fluorescence microscopy reveals that the DAPI staining results of the transient transformation are consistent with the green fluorescent signal, indicating that *PeWRKY31* localizes specifically to the nucleus.

### Resistance analysis of *PeWRKY31-*overexpressing tobacco

In recent years, with increasing number of studies on transcription factors, WRKY transcription factors have been found to be involved in a wide variety of biological processes in plants. Although *WRKY* genes have been studied in *Arabidopsis*, rice, and other model plant species [8, 9], there have been few studies on *WRKY* genes in poplar.

Overexpression of genes that respond to salt stress can improve salt tolerance in plants [36–38]. Under salt stress, the contents of antioxidant enzymes increase in chrysanthemum overexpressing *DgWRKY5*, whereas the malondialdehyde content decreases [39]. Antioxidant enzyme activities in transgenic wheat overexpressing *TaWRKY44* were much higher than in WT [22]. The soybean gene *GmWRKY12* responded to salt stress, and the MDA content decreased in *GmWRKY12*-overexpressing soybean [40]. *TaWRKY10*, *TaWRKY44*, and *TaWRKY19* in wheat responded to salt stress, and improved the tolerance of transgenic plants to salt stress [21, 22, 41]. In this study, a *PeWRKY31* overexpression vector was constructed and transformed into tobacco. Under salt treatment, the SOD and POD activities of *PeWRKY31*-overexpressing tobacco were significantly higher than those in WT, and the MDA content and electrical conductivity were significantly lower than that in WT. These results align with those of previous studies, suggesting that *PeWRKY31* could enhance the salt tolerance of tobacco.

*AtWRKY18*, *AtWRKY40*, and *AtWRKY60* in *Arabidopsis* belong to the WRKY IIa + IIb subfamily, and cooperate to regulate ABA-mediated salt stress responses [42, 43]. In *Nicotiana attenuate*, *WRKY3* and *WRKY6* respond to chew damage inflicted by herbivorous insects. WRKYs can regulate the transcription level of certain genes by activating signal pathways, such as those involved in salicylic acid (SA), ethylene (ET), and jasmonic acid (JA) [44–46]. Overexpression of *AtWRKY18-AtWRKY40* or *AtWRKY18-AtWRKY60* was found to regulate immunity to insects by regulating the JA and SA pathways in *Arabidopsis* [23]. AtWRKY28 and AtWRKY75 may enhance the resistance of *Arabidopsis* to fungal infection through the JA and ET pathways [47]. Overexpression of *OsWRKY53* in rice positively controlled resistance to certain insects by activating H_2_O_2_ and inhibiting the synthesis of ethylene [24]. All of these results indicate that WRKYs regulate susceptibility and resistance to insect pests by participating in signal transduction. In this study, *Populus × euramericana PeWRKY31* was upregulated after being subjected to biological stress, and overexpression of *PeWRKY31* in tobacco increased its resistance to insects. However, additional experiments are needed to determine whether *PeWRKY31* functions in *Populus* in the same manner as in tobacco.

### Transcriptome analysis

In the present study, the transgenic tobacco overexpressing *PeWRKY31* showed enhanced responses to biotic and abiotic stresses. Transcriptome KEGG enrichment analysis revealed differentially expressed genes were related to plant hormone signal transduction, plant MAPK signaling pathways, carotenoid biosynthesis, terpenoid and ketone biosynthesis, and other important salt-tolerance and insect-resistance pathways [44, 45]. Differentially expressed genes were frequent in glutathione metabolism, plant hormone signal transduction, and plant MAPK signaling pathways, all of which responded to biotic and abiotic stresses. *GSH-Px* catalyzed the conversion of reduced glutathione (GSH) to oxidized glutathione (GSSG), which scavenged harmful free radicals, thus protecting the structure and function of cell membranes against damage by peroxides [48]. Upregulated *APX* expression could enhance the ability of plants to tolerate oxidation, which in turn enhanced their ability to resist stress [49]. Upregulated expression of *LAR1* could enhance the salt tolerance and insect resistance of plants [48]. Upregulated expression of *JAR1* would increase JA-Ile, which promoted the formation of the COI1-JAZ co-receptor complex [50]. This ultimately led to the degradation of the JAZ protein. JAZ degradation can enhance the interaction between MYC2 and other proteins, activating the expression of the promoter target of MYC2 and enhancing the salt tolerance and insect resistance of tobacco [50]. The upregulated expression of *LAR1*, *APX*, *MYC*, and MAPK family genes in these pathways likely play an important role in regulating the responses of *PeWRKY31*-overexpressing transgenic tobacco to diseases, insect pests, and salt stress.

Transgenic tobacco lines differ in their resistance to biotic and abiotic stresses, which may be related to differences in the position of the *PeWRKY31* insertion; such differences may also regulate the expression of other DEGs. Downregulated expression of the homologous *NtWRKY31* gene in tobacco may be due to the inhibitory effect of overexpression of *PeWRKY31.* In future research, we will explore the regulatory mechanism of *PeWRKY31*, and provide a theoretical basis for breeding resistant poplars.

## Conclusions

In this study, *PeWRKY31* was isolated from *Populus × euramericana*, and its functions were investigated. Overexpression of *PeWRKY31* improved the resistance of transgenic tobacco to salt stress and pest stress. These results not only reveal the roles of *WRKY31* in *Populus × euramericana*, but also inform the use of *WRKYs* in future woody plant research. Future projects will include the overexpression of *PeWRKY31* in poplar, a model woody plant, to clarify its specific role in responses to biotic and abiotic stresses. These studies will have a goal of the generation of stress-resistant lines, with potentially great economic benefit.

## Methods

### Materials

The plant materials used were vegetative propagule of *Populus × euramericana* and wild-type (WT) tobacco (*Nicotiana tabacum* L.). The bacterial strains used were *Escherichia coli* DH10B and *Agrobacterium tumefaciens* GV3101. The plasmids pUCm-T, pCAMBIA1302, and pBI121 were used for vector construction. The materials were stored in Hebei Key Laboratory of Forest Germplasm Resources and Forest Protection, College of Forestry, Hebei Agricultural University.

### Quantitative reverse transcriptase–polymerase chain reaction

To analyze *PeWRKY31* expression in *Populus × euramericana* under different stresses and in different overexpressed tobacco lines, RNA was extracted from leaves and reverse-transcribed into cDNA. The purity of the RNA was determined based on the OD_260_/OD_280_ and OD_260_/OD_230_ ratios. A pair of specific primers (Table S1) and SYBR Green Master Mix (Roche) were used for qRT–PCR amplification of *PeWRKY31*. Eight DEGs were also screened for qRT–PCR based on transcriptomic data (for primer sequences see Table S1). Three independent biological experiments were performed, and actin and 18S were used as internal reference. Fold changes in expression levels were calculated by the ΔΔCT method (fold change = 2 − [ΔΔCT]). Real-time RT–PCR reactions were performed with initial incubation at 95 °C for 3 min, followed by 40 cycles of 30 s at 95 °C and 30 s at an annealing temperature of 55 °C.

### Isolation of *PeWRKY31*

Total RNA was extracted from leaves using an RNA extraction kit (Sainuo Biotech, Zhangjiakou, China), and the gel electrophoresis method was used to analyze the quality of the RNA. First-strand cDNA was synthesized by reverse transcription.

Transcriptome data were used to design primers to perform a screen for *WRKY* genes, resulting in the isolation of the *PeWRKY31* cDNA. The program Primer ver. 5.0 was used to design the specific primer set 31#all F/R based on the cDNA sequence; these primers were used for PCR amplification of the target gene from genomic DNA to isolate the full-length *PeWRKY31* gene. The PCR cycles used were: an initial 95 °C for 5 min, followed by 30 cycles of 95 °C for 30 s, 59 °C for 40 s, and 72 °C for 2 min. The target fragment was ligated into pUCm-T, and then transformed into *E. coli* DH10B competent cells by heat shock. The plasmid inserts of positive clones with ampicillin resistance were sequenced. The clone selected for further study was named as 31#all+T (Table S1 for primer information).

### Analysis of *PeWRKY31* sequence

The coding sequence of *PeWRKY31* was inferred using ORFfinder (https://www.ncbi.nlm.nih.gov/orffinder/) at the NCBI web site. The molecular mass and isoelectric point of PeWRKY31 were analyzed with ExPASy (http://web.expasy.org), and its secondary and tertiary structure were predicted using SOPMA (https://npsa-prabi.ibcp.fr/cgi-bin/npsa_automat.pl?page=/NPSA/npsa_sopma.html) and SWISSMODEL (https://www.swissmodel.expasy.org/interactive/DTDUCp/models/), respectively. The sequence was analyzed for potential nuclear localization signals using cNLS Mapper (http://nls-mapper.iab.keio.ac.jp/cgi-bin/NLS_Mapper_form.cgi), transmembrane regions using TMHMM (http://www.cbs.dtu.dk/services/TMHMM), signal peptides using seqNLS (http://mleg.cse.sc.edu/seqNLS/), and phosphorylation sites using NetPhos ver. 3.1(http://www.cbs.dtu.dk/services/NetPhos/) [26]. Searches for genes homologous to *PeWRKY31* were conducted in the NCBI and Phytozome databases. The predicted amino acid sequences of PeWRKY31 and homologous genes were compared using DNAMAN, and a molecular phylogenetic tree was constructed using the neighbor-joining method in MEGA ver. 7.0.

### Construction of GFP fusion subcellular localization vector and overexpression vector

Using the 31#all+T clone as a substrate, the primer sets 31#subl-orf F/R and 31#op-orf F/R were designed to amplify the predicted *PeWRKY31* coding sequence. The expression vectors p31#op+ 121and p31#subl+ 1302 were constructed by double enzyme digestion. The GFP fusion expression vector p31#subl+ 1302 and the overexpression vector p31#op+ 121 were transformed into competent *Agrobacterium tumefaciens* GV3101 cells by heat shock. The p31#subl+ 1302 insert region was amplified using the p31#orf F/R and hpt2jc F/R primer sets to verify the correct conformation of *PeWRKY31* and *GFP* in the vector in *Agrobacterium tumefaciens*, and likewise the p31#op+ 121 insert region was amplified with the p31#orf F/R and 103# F/R primer sets to verify the correct insertion of *PeWRKY31* and *nptII*.

### Subcellular localization analysis of *PeWRKY31*

The GFP fusion expression vector and the corresponding vector containing only the *GFP* gene insert were transiently transformed into WT tobacco leaves by agrobacterium infection. The leaves were observed for fluorescence signal using fluorescence microscopy to determine the subcellular location of GFP: *PeWRKY31* fusion protein.

### Genetic transformation of tobacco and identification of transgenic lines

*Agrobacterium* cells containing the overexpression vector p31#op+ 121 were oscillated in liquid LB medium containing kanamycin until reaching an OD_600_ of 0.4–0.6. Vigorous tobacco vegetative propagule were selected as transformation material. Leaves from the vegetative propagule were cut into sections of approximately 1 cm^2^, and then immersed in infection solution for about 10 min. The leaf discs were blotted on sterile paper, placed onto MS medium, and cultured in darkness for 36 h. The discs were then transferred to screening medium (MS medium with 2.0 mg L^− 1^ 6-BA, 0.1 mg L^− 1^ IBA, 800 mg L^− 1^ cefatoxime, and 50 mg L^− 1^ kanamycin) for further culture. After 30–50 days of selection, resistant vegetative propagules were transferred to rooting medium (MS medium with 800 mg L^− 1^ cefatoxime and 50 mg L^− 1^ kanamycin) for further rooting screening. Vegetative propagules with roots that had reached 2 cm in length were transferred to plastic pots containing a 3:1:1 mixture of peat: perlite: vermiculite and then grown in greenhouse. After 30 days, the fourth leaf was excised from plants exhibiting sufficient growth, and its DNA was extracted using a CTAB-based method. Two pairs of specific primers, 103#F/R and 35#35sendF with 31#-JCR, were used for confirmation of transgenic lines by PCR. For PCR controls, a plasmid containing the target gene was used as a positive control (CK+), and DNA from WT tobacco was used as a negative control (CK-).

### Salt stress treatment and physiological index determination of transgenic plants

For salt stress treatments, WT tobacco and three transgenic lines (B5, B12, A12) were planted in the same plastic pots in soil. The soil was treated with water containing either 0‰ or 4‰ NaCl, and the plants were grown in greenhouse. Both the treatment and the measurement of the indicators below involved three biological replicates.

After 72 h, specimen leaves of each treatment group were harvested to determine the peroxidase (POD) and superoxide dismutase (SOD) activities, malondialdehyde (MDA) content, and electrical conductivity. POD activity was determined using a method that quantified oxidized guaiacol. SOD activity was determined spectrophotometrically based on inhibition of the photochemical reduction of nitroblue tetrazolium. The free MDA content was determined using a thiobarbituric acid method. The relative electrical conductivity was measured using soaking method.

On days 0, 5 and 10 of salt stress, the plant height and ground diameter of the three transgenic lines (B5, B12, A12) and the WT were measured, and increases were calculated.

### Helicoverpa armigera feeding experiment

Leaves were excised from the WT and transgenic tobacco lines (B5, B12, A12) grown in soil for 30 days. The leaves of the same weigh were respectively placed in petri dishes with 15 and 8 *Helicoverpa armigera* (cotton bollworm) and each was repeated three times. The loss rates of leaf ingestion and infestation were recorded for each line.

### Transcriptome sequencing

Leaves of 30-day-old WT and transgenic (line B12) tobacco were sent to Tianjin Nuohe Zhiyuan Biological Company for transcriptome sequencing, and three biological replicates of each sample were used for the RNA-Seq. The differential gene expression was evaluated and analyzed by GO (http://geneontology.org/). The KEGG (https://www.ncbi.nlm.nih.gov/pmc/articles/PMC102409/) database was used to analyze the pathway enrichment of the differentially expressed genes [45]. The biochemical, metabolic, and signal transduction pathways related to the differentially expressed genes were recorded.

## Supplementary Information

**Additional file 1: Table S1**. The list of primers.

**Additional file 2: Fig. S1.** Predicted structure of *PeWRKY31*. a: Secondary structure of *PeWRKY31*. Blue, alpha helix; green, beta turn; red, extended strand; purple, random coil. b: Predicted tertiary structure of *PeWRKY31*.

**Additional file 3: Fig. S2.** Prediction of transmembrane structure, signal peptides, and phosphorylation sites on PeWRKY31. a: Prediction of transmembrane structure. b: Prediction of signal peptides. c: Prediction of phosphorylation sites.

**Additional file 4: Fig. S3.** Structures of plant transformation vectors. a: Subcellular localization vector p31#subl+1302. b: Overexpression vector p31#op+121.

**Additional file 5: Fig. S4.** DEGs and GO functional classification of differential genes in *PeWRKY31*-overexpressing transgenic and WT tobacco. a: Venn diagram showing the number of DEGs. Yellow represents the DEGs specific to B12, purple represents WT, and orange represents the DEGs common to the two strains. b: Vocano diagram showing the number of DEGs. Red is the up-regulated DEGs, and green is the down-regulated DEGs. c: GO functional classification. BP: Biological process; CC: Cellular component; MF: Molecular function.

**Additional file 6.**

## Data Availability

All data and materials are presented in the main paper and additional supporting file. All raw sequence data are available in the National Center for Biotechnology Information (NCBI) website with the SRA accession number of PRJNA694343 (https://www.ncbi.nlm.nih.gov/sra/PRJNA694343).

## Abbreviations

ORF: Open reading frame
TF: Transcription factor
KEGG: Kyoto Encyclopedia of Genes and Genomes
GO: Gene ontology
MAPK: Mitogen-activated protein kinase
GFP: GreenFluorescent Protein
SOD: Superoxide dismutase
POD: Peroxidase
MDA: Malondialdehyde
WT: Wild type
PCR: Polymerase Chain Reaction
