# Supplementary material for: Cloning and overexpression of PeWRKY31 from Populus × euramericana enhances salt and biological tolerance in transgenic Nicotiana
Source: BMC Plant Biol. 2021 Feb 6;21:80. doi: 10.1186/s12870-021-02856-3 (PMC7866765; doi:10.1186/s12870-021-02856-3)
Supplement: Supplementary file 1 — Additional file 1: Table S1. The list of primers. [file 12870_2021_2856_MOESM1_ESM.docx]

Table S1. The list of primers

| Primer name | Primer sequence（5’-3’） |
| --- | --- |
| PeWRKY31RT- F | TCGTTTATATCTCCAGCTACACC |
| PeWRKY31RT- R | TGAAATCCGATTCCAGATGCT |
| 18S F | TTAACGAGGATCCATTGGAGGGCA |
| 18S R | ACCCAACCCAAAGTCCAACTACGA |
| 31#all F | GCAAACCCTCCTCTCTCTCCCTCTA |
| 31#all R | GGAGACCATAGAAAGATAATTTAATAT |
| 31#orf F | ATGGCCAAAGGAAGTGGTGGACTCT |
| 31#orf R | TTAATCGCTCGGAAAACTTGAATTG |
| 31# subl-orf F | GGTCTCCcATGGCCAAAGGAAGTGGTGGA |
| 31# subl-orf R | GGTCTCAGATCTATCGCTCGGAAAACTTGAATTG |
| 31#op-orf F | TCTAGAACCATGGCCAAAGGAAGTGGTGGA |
| 31#op-orf R | GAGCTCTTAATCGCTCGGAAAACTTGAATTG |
| M13# F | GTTGTAAAACGACGGCCAGT |
| M13# R | CAGGAAACAGCTATGACCATGA |
| 35#35send F | ATTTCATTTGGAGAGAACACGG |
| 31#-JC R | TCCAATCCAGCGGGACTTCCAGA |
| hpt2jc F | GCTCCATACAAGCCAACCAC |
| hpt2jc R | GAAAAAGCCTGAACTCACCG |
| 103# F | ATCTCCTGTCATCTCACCTTGCTCCT |
| 103# R | TCAGAAGAACTCGTCAAGAAG |
| Nt-18S F | GCAAGACCGAAACTCAAAGG |
| Nt-18S R | TGTTCATATGTCAAGGGCTGG |
| Actin F | TGTGTTGGACTCTGGTGATG |
| Actin R | CGCTCGGTAAGGATCTTCATC |
| NtWRKY31RT- F | TACTGGTTTGCATCTTCTCACT |
| NtWRKY31RT- R | CTTCAGAGTTAGGGGATAAGCC |
| 1# F | ACCAATCTGTGAATCCCTCATT |
| 1# R | CTCTTGTAAGGATCAGAAGGCA |
| 2# F | TACTATGGGAAATTATGGGCCC |
| 2# R | GAGCATCCAGTCTCCATCTTTA |
| 3# F | CTATGATTGGCGCCGTTATAAC |
| 3# R | CAATTTTCGGGTCATTCTCTCC |
| 4# F | CCATCAAAACCAACACCAACAA |
| 4# R | CCAGCCATTGGGTAAAATGAAA |
| 5# F | GATCGAATCATGTCAGAAACCG |
| 5# R | TGAGGAAGCATTAGAGGAGTTG |
| 6# F | ATACGAAGAGGAAAGGACACAG |
| 6# R | AAGGTTAGCTAAGTACCACGAC |
| 7# F | TGGATCCAGAAATTGTGACTCA |
| 7# R | TCTCTAGCTGCACTCCATATTG |
| 8# F | CCTTCTGGTCTACTTTCACCTT |
| 8# R | AGTAATCCAACTCCAACTGTGT |
