# Supplementary figures and images for: Cloning and overexpression of PeWRKY31 from Populus × euramericana enhances salt and biological tolerance in transgenic Nicotiana
Source: BMC Plant Biol. 2021 Feb 6;21:80. doi: 10.1186/s12870-021-02856-3 (PMC7866765; doi:10.1186/s12870-021-02856-3)

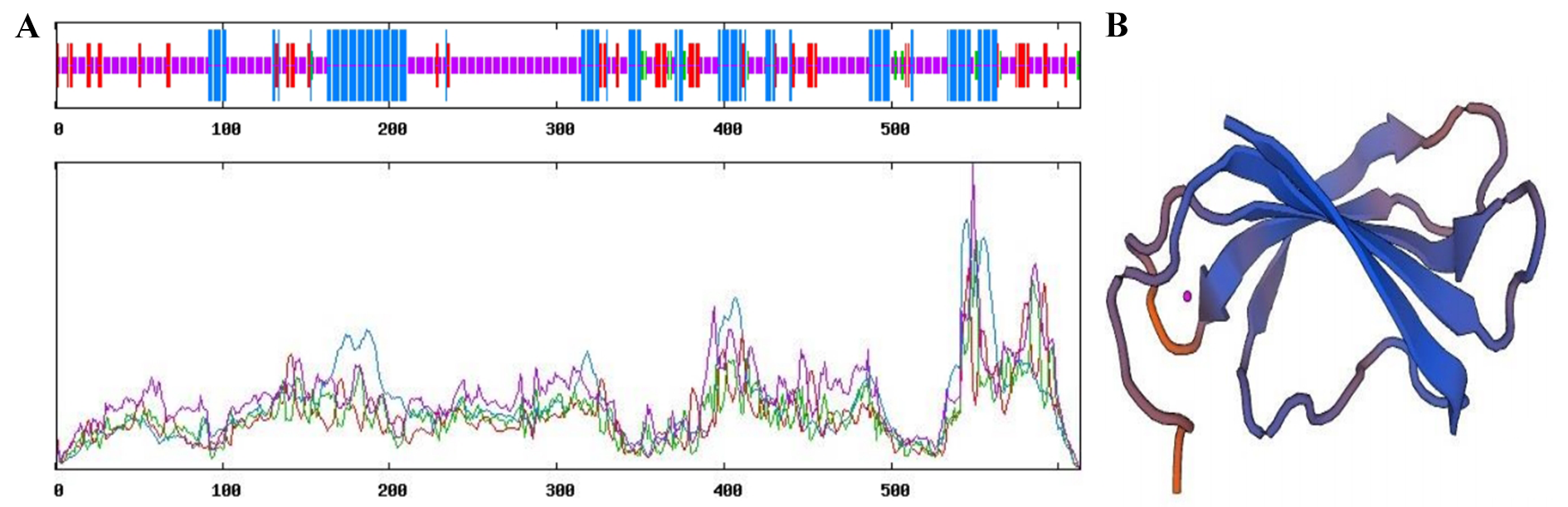

Supplement: Supplementary file 2 — Additional file 2: Fig. S1. Predicted structure of PeWRKY31. a: Secondary structure of PeWRKY31. Blue, alpha helix; green, beta turn; red, extended strand; purple, random coil. b: Predicted tertiary structure of PeWRKY31. [file 12870_2021_2856_MOESM2_ESM.tif]

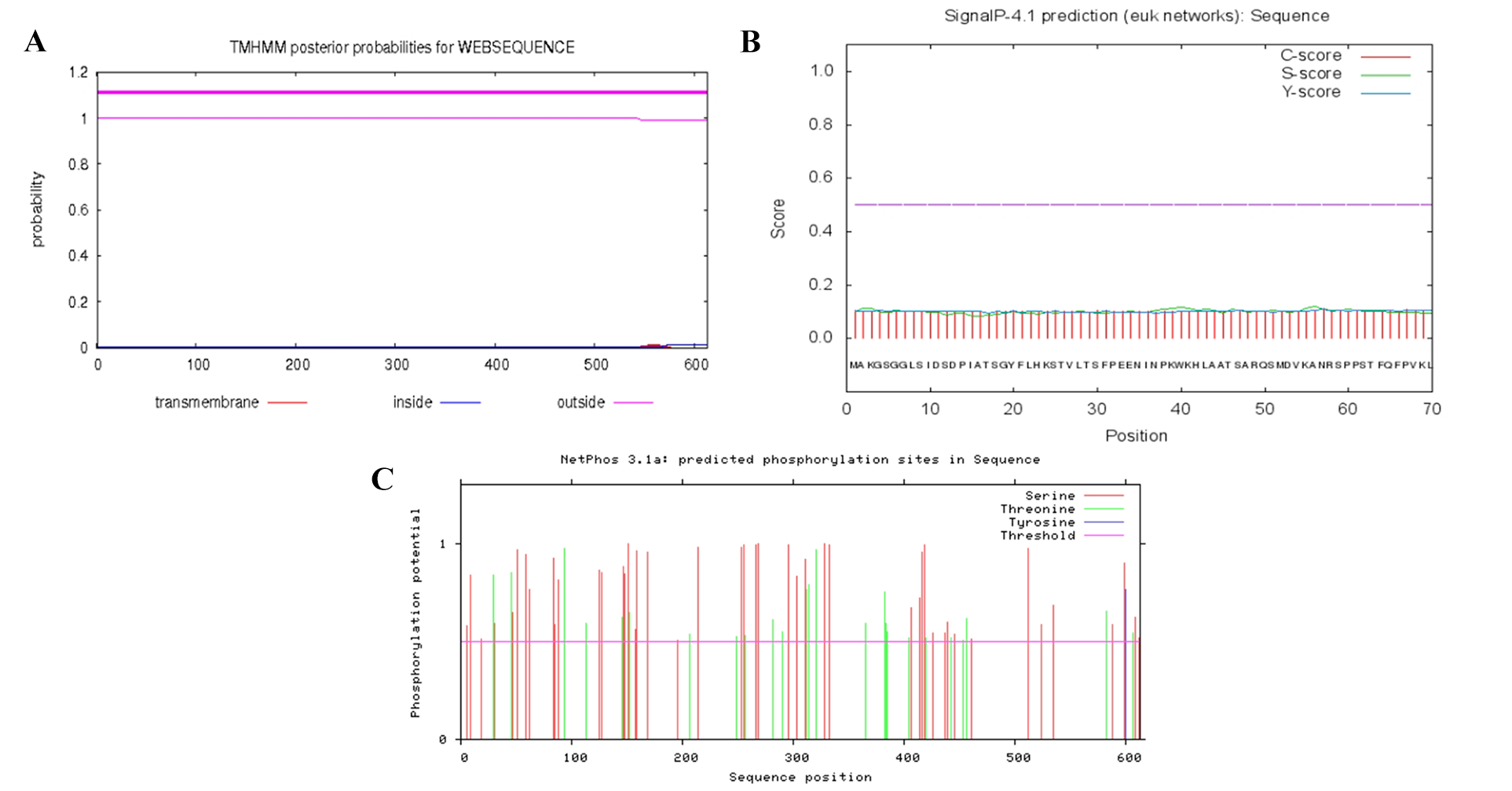

Supplement: Supplementary file 3 — Additional file 3: Fig. S2. Prediction of transmembrane structure, signal peptides, and phosphorylation sites on PeWRKY31. a: Prediction of transmembrane structure. b: Prediction of signal peptides. c: Prediction of phosphorylation sites. [file 12870_2021_2856_MOESM3_ESM.tif]

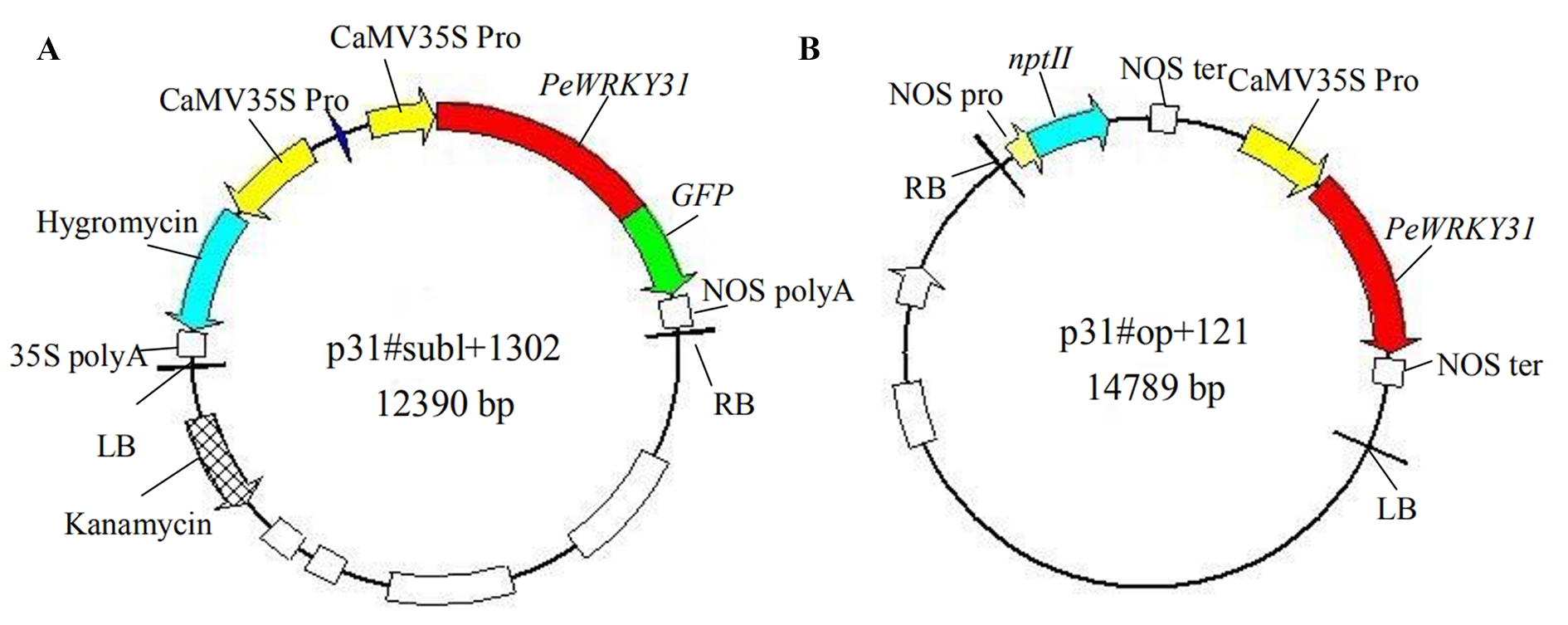

Supplement: Supplementary file 4 — Additional file 4: Fig. S3. Structures of plant transformation vectors. a: Subcellular localization vector p31#subl+1302. b: Overexpression vector p31#op+121. [file 12870_2021_2856_MOESM4_ESM.tif]

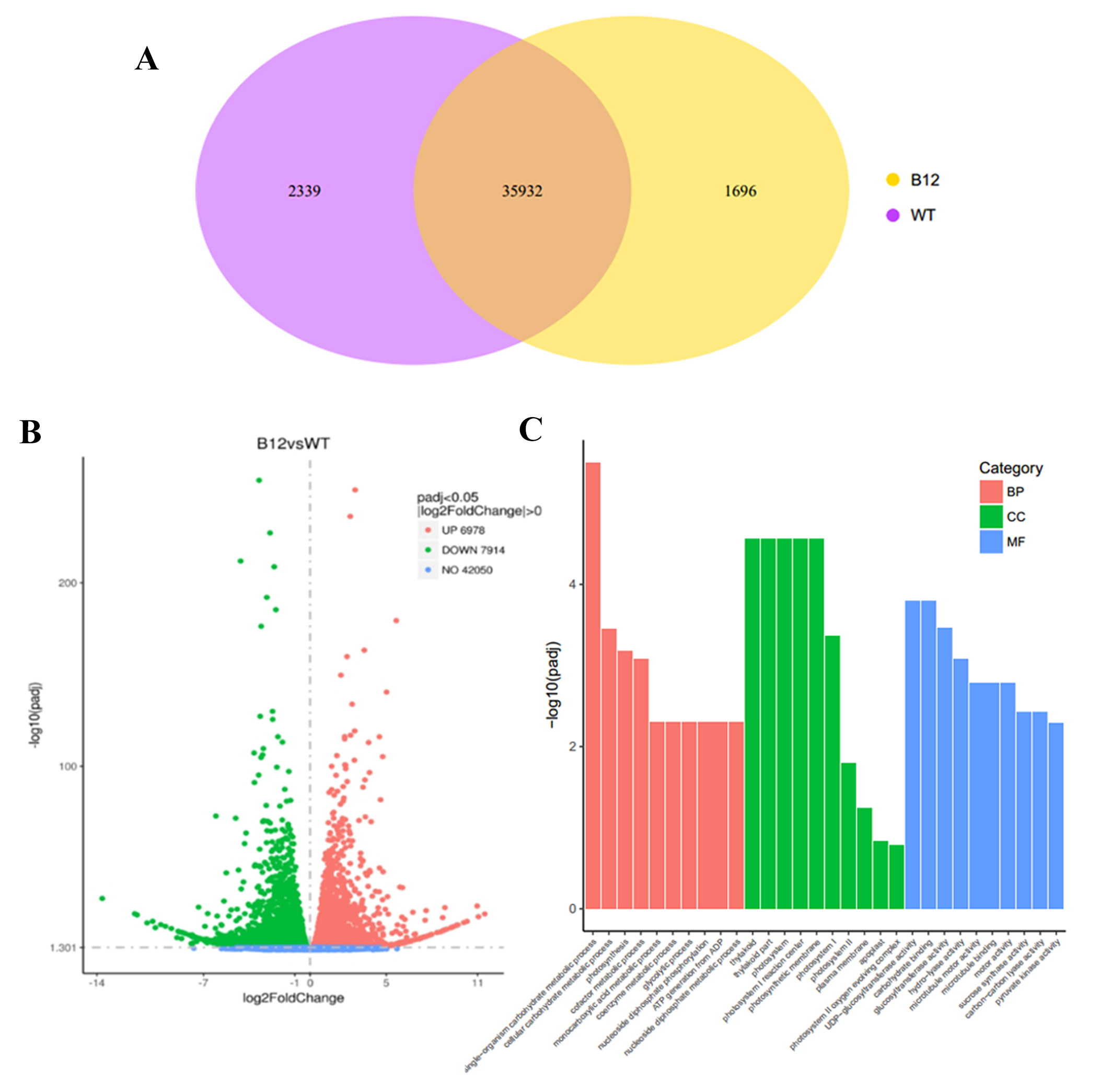

Supplement: Supplementary file 5 — Additional file 5: Fig. S4. DEGs and GO functional classification of differential genes in PeWRKY31-overexpressing transgenic and WT tobacco. a: Venn diagram showing the number of DEGs. Yellow represents the DEGs specific to B12, purple represents WT, and orange represents the DEGs common to the two strains. b: Vocano diagram showing the number of DEGs. Red is the up-regulated DEGs, and green is the down-regulated DEGs. c: GO functional classification. BP: Biological process; CC: Cellular component; MF: Molecular function. [file 12870_2021_2856_MOESM5_ESM.tif]

Full-length gel

1 2 3 4 M

M 1

1 1 2 2 3 3 M


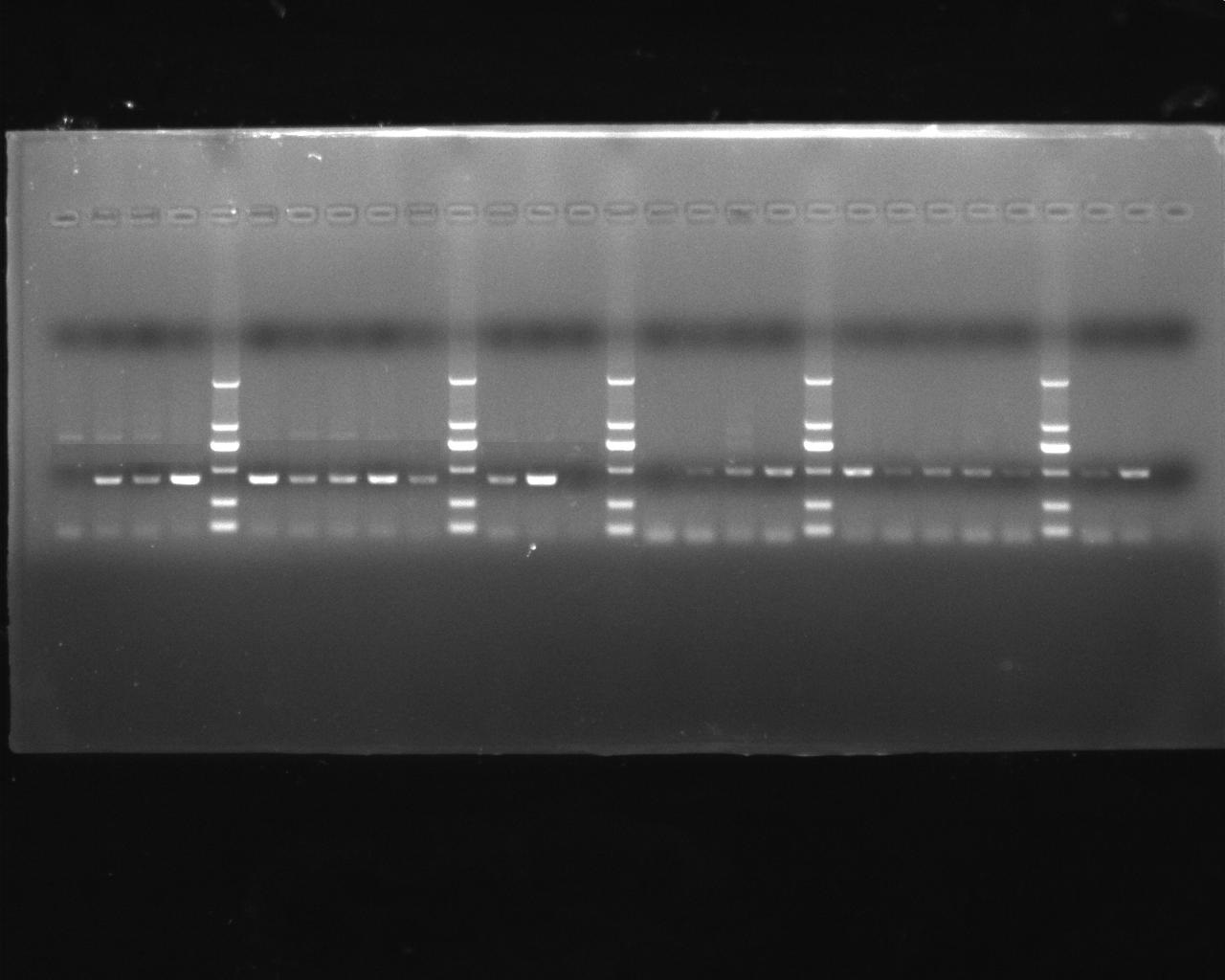


B1 B2 B3 B5 M B12 A6 A7 A12 A13 M A14 CK+ CK-

M B1 B2 B3 B5 M B12 A6 A7 A12 A13 M A14 CK+ CK-

Fig.4(E) and Fig.6(F)

Supplement: Supplementary file 6 — Additional file 6. [file 12870_2021_2856_MOESM6_ESM.docx]
